# Black soldier fly larvae meal supplementation in a low protein diet reduced performance, but improved nitrogen efficiency and intestinal morphology of duck

**DOI:** 10.5713/ab.23.0259

**Published:** 2023-11-02

**Authors:** Rinanti Eka Aldis, Muhlisin Muhlisin, Zuprizal Zuprizal, Heru Sasongko, Chusnul Hanim, Muhsin Al Anas

**Affiliations:** 1 Animal Nutrition and Feed Science Department, Faculty of Animal Science, Universitas Gadjah Mada 55281, Yogyakarta, Indonesia; 2 Animal Production Department, Faculty of Animal Science, Universitas Gadjah Mada 55281, Yogyakarta, Indonesia

**Keywords:** Ammonia, Black Soldier Fly Larvae Meals (BSFLM), Duck Performance, Low Protein Diet, Soybean Meal

## Abstract

**Objective:**

Reduced crude protein (CP) diets offer potential benefits such as optimized feed efficiency, reduced expenses, and lower environmental impact. The objective of this study was to evaluate black soldier fly larvae (BSFL) meal on a low-protein diet for duck performance, blood biochemical, intestinal morphology, gastrointestinal development, and litter.

**Methods:**

The experiment was conducted for 42 days. A total of 210-day-old male hybrid ducklings (5 replicate pens, 7 ducks per pen) were randomly assigned to 6 dietary treatments (3×2 factorial arrangements) in randomized design. The factors were CP level (18%, 16%, 14%) and protein source feed soybean meals (SBM), black soldier fly larvae meals (BSFLM).

**Results:**

Reduced dietary CP levels significantly decreased growth performance, feed intake, the percentage of nitrogen, pH (p<0.05), and tended to suppress ammonia in litter (p = 0.088); increased lipid concentration; and enhanced relative weight of gastrointestinal tracts (p<0.05). In addition, dietary BSFL as a source of protein feed significantly increased lipid concentration and impacted lowering villus height and crypt depth on jejunum (p<0.05).

**Conclusion:**

In conclusion, the use of BSFLM in a low-protein diet was found to have a detrimental effect on growth performance. However, the reduction of 2% CP levels in SBM did not have a significant impact on growth performance but decreased nitrogen and ammonia concentrations.

## INTRODUCTION

Protein feed ingredients are characterized by a relatively high cost, with soybean meal (SBM) being the predominant protein source in widespread utilization. The incorporation of SBM into animal feed typically constitutes a proportion of 23% to 30% of the overall feed composition [1]. Indonesia’s livestock feed industry relies mainly on imported SBM (over 5.3 million metric tons) to meet this demand [2]. The condition is challenging in managing feed prices, which have shown a tendency to steadily rise. As a result, there has been a focus on enhancing the efficiency of utilizing local feed as viable substitutes for conventional feed ingredients.

Considering this circumstance, contemporary research is focused on exploring alternative protein sources that are both sustainable and do not pose a threat to human food production. The application of black soldier fly larvae meal (BSFLM) has been recognized as a potential alternative protein, energy source in broiler chicken diets, more sustainable than soybean and does not compete with human food production [3,4]. They possess a high content of crude protein (CP) (55.42% to 65.50%) [5,6] and energy (7% to 39%) [7], along with a favorable amino acid (AA) composition, rich in calcium (1.21% to 4.39% dry matter [DM]), and phosphorus (0.74% to 0.95% DM) [8]. According to Gariglio et al [9] partially defatted BSFLM contains lauric acid (C12:0) 49.7% of the total fatty acids followed by palmitic acid (C16:0; 13.3%) and myristic acid (C14:0; 10.1%). Meanwhile, according to Rostagno et al [10], the standardized ileal digestible (SID) value of SBM is 44%, which is higher than BSFLM. According to Tansil et al [11], the SID value of partially defatted black soldier fly larvae (BSFL) was found to be 2.7%, slightly lower than SBM but 5% higher than fish meal. This is related to the chitin content in BSFLM, influenced by the age of the larvae. The BSFLM is composed of chitin and medium-chain fatty acids, including lauric and myristic acid. These components are believed to enhance intestinal and immunological health in broiler chickens due to their prebiotic and antibacterial effects [12,13].

On the other hand, insects such as BSFL not only have a high nutrient content, but also produce low greenhouse gas and ammonia emissions, have a good feed conversion ratio (FCR) as cold-blooded animals, and require little water and land to grow [14–16]. These conditions accord with a concerted initiative to optimize the utilization of CP in feed rations by implementing N-balanced diets based on easily digestible amino acids, multi-phase feeding, addition of essential amino acids and other feed additives [17]. Reducing feed protein by an average of 20 to 30 g/kg is an effective recommendation for sustaining animal performance and production. However, lowering CP by more than 30 g/kg has a detrimental effect on performance and leads to an increase adipose fat [18,19]. On the contrary, the reduction in dietary CP levels from 17.22% to 13.54% did not result in a decline in body weight gain (BWG), productivity, or feed consumption among ducks [20]. In another study, it was shown that the inclusion of crystalline amino acids with low dietary CP levels of 16% to 12% did not provide a significant impact on final body weight (BW), BWG, and feed intake (FI). However, a notable enhancement in FCR was seen [21].

Moreover, there is a limited amount of information regarding the application of BSFLM in low-protein diets in duck production. In Indonesia, ducks have emerged as a viable alternative for animal protein, as evidenced by a growing demand over time. Consequently, enhancing production efficiency becomes a crucial factor to consider. Hence, the aim of this research was to investigate the potential of BSFLM as a source of protein feed and determine the efficiency of CP utilization in duck diets. The findings will offer theoretical and technical support for using a low-protein diet in the duck industry.

## MATERIALS AND METHODS

### Animal care

The experiment was reviewed and approved by the research ethics committee Faculty of Veterinary Medicine, Universitas Gadjah Mada with the ethical clearance No: 024/EC-FKH/Eks./2023.

### Animals and housing

A total of 210 1-day-old male hybrid ducks (male pekin duck ×female hybrid duck) were reared for 42 days and fed with the same standard starter diet from d 1 to 10. Ducks were weighed on day 11 and randomly distributed with an initial BW of 217.7±1.3 g in 30 floor pens with a size of 1.1×0.6 m and 7 ducks per pen. Each pen was equipped with a feeder, a drinker, and rice hulls as litter material. Ducks had free access to feed and water throughout the experiment. The room temperature was maintained at 31°C in the first week and gradually reduced to 2.5°C after each week until 20°C. In the early stages of growth, lighting plans provide for a lengthy day with 23 hours of light and 1 hour of darkness for up to seven days. After seven days, 5 hours of darkness (4 to 6 hours) may be optimal.

### Black soldier fly larvae processing

The larvae used for this experiment were produced at the Greenprosa Adikara Company (Banyumas, Central Java) and obtained by processing larvae reared on vegetable by-product substrate. The BSFL were collected after they reached a developmental stage of 12 days. Subsequently, they underwent a series of procedures, including washing with hot water at 80°C for 5 minutes. Following this, the larvae were treated to drying using a rotary drier at 300°C for 10 minutes. The next step involved extraction using a hot extruder operating at 100°C. Finally, the larvae were processed into dish meal using a sieve with a mesh size of 0.5 mm. The BSFL samples were analyzed in a Laboratory of Animal Biochemistry, Faculty of Animal Science, Universitas Gadjah Mada (Indonesia) for DM, CP, lipid, fiber, and mineral analysis [22]. According to the analysis, the DM, protein, lipid, fiber, calcium, and phosphor content of the BSFLM were 95.63%, 54.92%, 8.99%, 8.38%, 2.51%, 0.18% (DM basis), respectively.

### Experimental design and diets

This research had a 2×3 factorial arrangement with 2 types of protein source feedstuff (SBM and BSFLM) and 3 protein levels (14%, 16%, 18%). Six diets consisted of i) SBM-18%, ii) SBM-16%, iii) SBM-14%, iv) BSFLM-18%, v) BSFLM-16%, vi) BSFLM-14% (Table 1). A total 6 treatments (5 pen replicates; 7 ducks/pen) randomly distributed to 30 floor pens. Dietary treatments were given to ducks from day 11 to day 42. Respectively diets and water were provided *ad libitum* from day 1 to 42. Diets were completed with synthetic AA (feed-grade) lysine, methionine threonine, tryptophan, arginine, valine, and isoleucine.

### Data collection

#### Growth performance parameters

Individual BW and FI in each pen were recorded on 21, 35, and 42 days to determine BWG and FCR. The mortality of the bird was recorded to adjust the feed-to-gain ratio. Two birds from each replicate with an average BW were weighed and slaughtered with the halal method, and one sample was collected from the gastrointestinal tract for measuring the development.

#### Blood biochemical profiles

On d42, one bird with an average weight was selected from each replicate (n = 6), weighed, and slaughtered by decapitation to collect blood serum, which was then preserved at a very low temperature of −20°C until analyzed. The total protein, albumin, glucose, total cholesterol, high density lipoprotein (HDL), low density lipoprotein (LDL), triglyceride, phosphate, and calcium concentrations were determined using a UV-visual photometer (Microlab 200: Merck Vital Scientific, Darmslandt, The Netherland) with commercial kits (DiaSys diagnostic System GmbH, D-65558 Holzheim, Germany).

#### Morphology of the jejunum measurement

Samples were fixed in 10% formalin prepared using the paraffin embedding procedures. Samples were sectioned at 2-μm cuts and stained with hematoxylin and eosin. A total of 30 intact, well-structured crypt-villi units were randomly selected and measured per sample. The villus height (VH) from the tip of the villus to the crypt opening and crypt depth (CD) from the base of the crypt to the level of the crypt opening) were determined using electron transmission microscope and digital camera (Optilab Advance, Miconos) for image processing. The structure of the jejunum can provide information into the intestinal capacity for nutrition absorption and consistently correlates with animal performance. The height of villi is a reliable measure of the gastrointestinal tract’s capacity to effectively uptake nutrients. The depth of crypts serves as a reliable measure of the level of maturation in the intestinal epithelium, with deeper crypts suggesting a more advanced stage of maturation. The ratio between VH and CD serves as an indicator of the overall functionality and efficiency of the intestine [23].

#### Gastrointestinal development measurement

The relative weight (g/kg BW), length (cm) of gastrointestinal tract and pH the intestinal segments (digesta), including the proventriculus, gizzard, duodenum (gizzard-the bile duct), jejunum (the bile duct-Meckel’s diverticulum), ileum (Meckel’s diverticulum-ileocecal junction) were collected and measured. Each gastrointestinal tract was cleaned from digesta before measured.

#### Litter composition

Litter samples were collected at four locations in each pen, avoiding the drinking area. These four samples were homogenized into one composite sample per pen. Litter samples were immediately kept at 20°C after collection. The standard analysis was used to determine the DM, organic matter, nitrogen concentration [22], and ammonia concentration [24].

### Statistical analysis

All experimental data were analyzed statistically using IBM SPSS statistic version 26.0. This model included the main effects of protein source feedstuffs, dietary CP levels, and their interaction. Data obtained were analyzed for normality by the UNIVARIATE procedure and using variance continued with Duncan’s Multiple Range Test (DMRT). The statistical significance of all analyses was set at p<0.05 for probability values.

## RESULTS

### Growth performance parameters

The effects of BSFLM supplementation and CP levels on FI, WG, and FCR are presented in Table 2. The significant effects of reducing dietary CP levels were to decrease FI, WG, and increase FCR (p<0.05). Similarly, to the effect of dietary CP levels, the factor of protein sources significantly affected growth performance. Soybean meal showed a better performance than BSFLM as a protein source feed (p<0.05). Furthermore, the data also showed that CP levels×protein source interaction on SBM-18% and SBM-16% treatments were not significant on WG and FCR (p>0.05).

### Blood biochemical profiles

The effects of BSFLM supplementation and CP levels on blood biochemicals is shown in Table 3. CP levels× protein sources interaction was significant (p<0.05) for total protein, cholesterol, LDL, HDL, albumin, and phosphate concentration. The reduction of 2% CP level on treatments SBM-18% and SBM-16% did not show a significant effect on biochemical profiles (p>0.05) except for HDL. CP level reduction in diet markedly increased triglyceride, cholesterol, LDL, HDL, albumin, and phosphate concentrations (p<0.05). Similarly, irrespective of dietary CP levels, dietary BSFLM as a source of protein feed significantly resulted in a higher concentration of cholesterol, LDL, and HDL than SBM as a protein source (p<0.05).

### Morphology of the jejunum measurement

The effects of BSFLM supplementation and CP levels on the VH, CD, VH/CD ratio of jejunum are presented in Table 4. There was an interaction between dietary CP levels and protein sources for VH (p<0.05). The SBM diet treatment resulted in a reduction in CP levels, which subsequently led to a drop in VH. Conversely, ducks that were fed BSFLM showed a significant rise in VH when their CP levels were lowered. In addition, using BSFLM resulted in lower VH and CD than using SBM as a protein source (p<0.05).

### Gastrointestinal development measurement

The interaction between dietary CP levels and protein sources (Table 5) is significant in the relative weight of the duodenum, ileum, caecum, large intestine, and length of the proventriculus (p<0.05). The reduction of CP levels by 2% in both SBM and BSFLM did not have a statistically significant effect on gastrointestinal development. A significant difference (p< 0.05) in CP level treatment was also noted for the relative weight of the gastrointestinal tract, the higher the reduction in CP level, the greater the enhancement of the gastrointestinal tract weight (p<0.05). There was a significant difference (p>0.05) in the relative weight of jejunum and the length of gizzard due to protein source treatment.

### Litter composition

The effects of BSFLM supplementation and CP levels on litter composition are shown in Table 6. Factor of dietary CP levels significantly decreased the percentage of nitrogen and pH of the litter (p<0.05) and tended to decrease ammonia in the litter (p = 0.088). Regardless, there was no significant effect on CP levels×protein source interaction. Similarly, the protein source factor was also found to have no significant impact on litter composition (p>0.05).

## DISCUSSION

The advantages of lowering protein levels in poultry diets while supplying AA requirements have been reported in several studies of poultry performance [25,26]. However, in the present study, meeting AA requirements such as methionine, lysine, threonine, arginine, valine, isoleucine, and tryptophan on a low-protein diet resulted in linearly depressed growth performance (p<0.05), regardless of various protein sources. The lowering CP levels affected to reduce the growth performance, as also reported by Wang et al [27], the result was validated by the data, where reduction CP levels suppressed the FI, BW, weight gain, and ADG and increased FCR. Wang et al [28] in their research stated that a decrease in feed protein levels (13.5% and 15.5%) reduced FI and BW in ducks aged 15 to 35 days. The suppression of growth performance could be associated with lower levels of non-essential amino acids such as glycine, serine, or glutamine [29] and feed ingredient digestibility [28]. This was reported by Widyaratne and Drew [30] that applying a low-protein diet to poultry could affect growth performance at similar levels to high-protein diets when using highly digestible feed ingredients. In other research by Wang et al [28] stated that a high protein level decreased growth performance due to increased levels of lower digestible ingredients. In the present study, the interaction between the main factors showed BSFLM-14% as the lowest growth performance. This related to the low passage rate and length of time of feed in the gastrointestinal tract, which were associated with nutrient content and feed particles [31]. It is interesting to note that a reduction of 2% in CP levels in SBM as a protein source did not have a significant impact on the growth performance of ducks. This finding was in line with the results of studies by Xie et al [20] and Sigolo et al [25], which found that adding crystalline amino acids decreased CP levels by 2.5% to 3.68% without affecting chicken productivity. On the other hand, the treatment of BSFLM protein sources affected growth performance and FI. Thus, the decrease in productivity could be associated with the chitin content of BSFL (6.67%) which influenced digestibility value and feed transit duration in the digestive tract [31,32].

The data showed that descending CP levels in a diet increased the concentration of triglycerides, lipids, albumin, and phosphate. This was confirmed by Law et al [33] in their study, which indicated a rise in triglycerides, albumin, free fatty acids [33,34], cholesterol, LDL, and HDL in the blood [31]. The significant rise in these parameters was due to an increase in the proportion of energy sources in the feed, which was related to the process of lipogenesis in the liver. Kamran et al [35] also confirmed that dietary CP levels and *in vitro* lipogenesis were inversely linked. The higher dietary CP content decreased glucose utilization and increased its synthesis from substrates that were used for fat synthesis previously. Kamran et al [35] stated that birds with low protein diets would use carbohydrates as an energy source rather than free fatty acids, showing greater triglycerides in their blood plasma. This would also affect the level of LDL that is a lipoprotein responsible for transporting triglycerides from liver via the blood to extrahepatic tissue. In the present study, the dietary BSFLM on ducks increased the concentration of lipid and decreased albumin and phosphate levels. This related to BSFL consisting of 52% saturated fatty acid in the form of lauric acid, which stimulates cholesterol synthesis in the blood by raising the activity of enzymes involved [36]. In our research, the treatment of CP levels and protein sources resulted in a total protein in serum between 2.76 to 3.38 g/dL, with the lowest shown on BSFL-14%. According to Behera et al [37], the total blood protein of 8-week-old ducks is between 6.3 to 6.4 g/dL in the treatment of reduced protein levels up to 4%.

The longer the villi, the greater the value of nutritional absorption. Decreasing villus height leads to a lower intestinal absorption area, therefore, gut morphology is crucial in determining the efficiency of dietary nutrients [31]. In this present study, the interaction between dietary CP levels and protein sources markedly on VH. Lowering CP on BSFLM dietary levels had higher VH compared with higher CP levels, this result agreed with Shazali et al [38] but this contradicted with using SBM on low CP. Gu and Li [39] reported that increasing CP levels affected a higher number of goblet cells in the distal jejunum that was associated with VH, CD, and epithelium cell size. Interestingly, decreasing 2% CP levels (SBM-18% to SBM-16%) did not show a significant effect on jejunum morphology, thus indicating that decreasing CP levels could be carried out by supplying synthetic amino acids. In the current study, applying BSFL showed lower VH and CD than SBM as a protein source (p<0.05). Jiang et al [31] stated that the height of the villi and CD were mostly determined by diet. This is related to the digestible value of each protein source, SBM has a higher digestibility value (SID 44%) than BSFL [10]. According to Tansil et al [11], SID-partially defatted BSFL was 2.7% lower than SBM but 5% higher than fish meal. The variation in digestibility influenced the availability of amino acids, which are essential for villus development. The supply of non-essential amino acids such as glycine, glutamine, and proline contributed to the development of intestinal villi [33]. In their study, Wang et al [27] discovered that a decrease in essential amino acids, particularly free branched chain amino acids, damaged intestinal morphology. This was supported by the findings of Facey et al [32], who discovered that low concentrations and digestibility of proteins had a negative impact on development and function of the intestine. Depending on the protein source, the percentage of undigested CP in various poultry feedstuffs ranges from 8% to 35% [40]. Interestingly, the dietary CP levels markedly enhanced the relative weight of the gastrointestinal tract of ducks (p<0.05) when the CP levels were reduced. In contrast, the length of the proventriculus, gizzard, and large intestine significantly decreased as a result of reducing CP levels. The development of gastrointestinal tract (GIT) was related to amino acids supplied in the starter phase as energy sources and building blocks, especially for the small intestine. It has been shown that quality and AA levels can affect gene expression of transporter proteins in the small intestine. Thus, suboptimal AA (protein quality) suppressed gut function. The optimal AA supplied in the starter diet will improve GIT development and its capacity for digestion and nutrient absorption [41]. In contrast, in the present study, lowering CP levels with AA supplementation showed improved weight of small intestinal organs. On the other hand, this result related to the improvement of feedstuffs proportion and particle formation. The higher the reduction of CP levels, the more corn is added to achieve the energy requirement. Furthermore, decreasing 2% CP on the SBM diet had no marked effect on gastrointestinal tract development except for jejunum and ileum weight. According to Kleyn and Chrystal [42], increasing the feed particle size had a positive impact on gizzard development (as much as 5%) by optimizing size, function, and nutrient absorption. In addition, large particles, such as in whole grains, stimulated 44% of relative gizzard weight [43], decreased pH, improved proventriculus dilatation, digestibility, and starch retention [44]. The particle structure and fiber components of feed played a function in modulating the passage rate of digesta through the GIT [31]. Coarse feed particles will remain in the gizzard for a longer period, resulting in greater muscle activity and gizzard weight [45]. According to Auza et al [46], providing 11.25% BSFL flour had a substantial effect on expanding the length of the ileum, this was related to chitin component [47]. Furthermore, the presence of antimicrobial peptide in BSFL contributed to the development of gastrointestinal tract and inhibited pathogenic microbes in the gut, hence enhancing gut balance [48].

Decreasing the CP levels in the diet reduced the percentage of nitrogen, ammonia (p = 0.088) and pH of litter (p<0.05). It is well-documented that decreasing dietary CP levels (1%) reduced nitrogen excretion (about 10%) [49] and litter moisture [50]. Collin et al [51] reported that most N losses through excreta were caused by a dietary CP inability to meet AA requirements, notably imbalances between AA. This had a significant impact on poultry metabolism and endocrine functioning. In addition, reducing dietary CP levels had a correlation with enhancement of animal proportion without lesions [52], decreased water consumption and ammonia concentration (acidic fluids) [53] as well as heat increment [54]. This condition affected the pH level, the lower the pH, the less unionized ammonia is available for volatilization. Ammonia concentration tended to rise with increasing moisture content of the litter due to the majority of microbe species relying on water for proliferation [55]. Normally, the pH of litter ranges between 6.5 and 8.5 [55], a pH below 7.0 (neutral) declined the uricolytic bacterial population is responsible for the production of ammonia and the resulting growth of other bacteria that absorb ammonia. This process leads to a reduction in the volatilization of ammonia in the environment around it [26].

## CONCLUSION

In conclusion, supplementation with BSFLM in low-protein diets declined duck performance, reduced nitrogen, and ammonia in the litter, increased the morphological size of the jejunum, digestive tract, and blood lipid concentration. In contrast, the reduction of 2% CP in SBM-treated diets did not affect growth performance but decreased nitrogen and ammonia concentrations. Thus, the supplementation of BSFLM on low-protein duck diets must consider the stage of larvae related to the digestibility value.

## Figures and Tables

**Table 1:** Ingredient and nutrient composition of experimental diets

Items	SBM (%)			BSFLM (%)		
	
	18	16	14	18	16	14
Ingredients (%)						
Corn	66.38	72.29	75.84	69.50	72.00	74.49
Rice bran	10.00	12.00	14.50	12.00	13.10	15.00
Soybean meal	15.00	7.50	1.00	0.00	0.00	0.00
BSFL	0.00	0.00	0.00	10.00	7.00	3.00
Meat bone meal	5.00	4.00	3.00	4.00	2.50	1.00
Crude palm oil	1.00	0.00	0.00	0.00	0.00	0.00
Limestone (CaCO_3_)	0.70	0.90	0.95	0.85	1.10	1.30
Di-Calcium phosphate	0.40	0.60	0.95	0.85	1.00	1.30
Salt	0.31	0.31	0.31	0.31	0.31	0.31
Vitamin^1)^	0.03	0.03	0.03	0.03	0.03	0.03
Mineral premix	0.15	0.15	0.15	0.15	0.15	0.15
DL-Methionine	0.17	0.25	0.33	0.28	0.31	0.35
L-Lysine HCl	0.14	0.44	0.70	0.45	0.58	0.73
L-Threonine	0.07	0.23	0.37	0.24	0.30	0.39
L-Arginine	0.00	0.26	0.49	0.34	0.44	0.55
L-Valine	0.00	0.17	0.31	0.12	0.21	0.32
L- Isoleusine	0.00	0.17	0.33	0.20	0.26	0.34
L-Trypthopan	0.05	0.10	0.14	0.09	0.11	0.14
Choline chloride	0.10	0.10	0.10	0.09	0.10	0.10
Toxin binder	0.20	0.20	0.20	0.20	0.20	0.20
Sodium carbonat	0.30	0.30	0.30	0.30	0.30	0.30
Total	100.00	100.00	100.00	100.00	100.00	100.00
Analyzed nutrients						
Dry matter (%)	89.54	88.87	89.21	89.66	89.57	88.95
Gross energy (Cal/g)	3,967.77	3,883.80	3,904.76	3,814.26	3,835.89	3,825.24
Crude protein (%)	17.60	16.64	14.78	17.55	16.67	14.52
Calculated nutrients (%)						
Lysine	0.93	0.93	0.93	0.93	0.93	0.93
Methionine	0.44	0.48	0.53	0.52	0.53	0.54
Cystine	0.30	0.25	0.21	0.22	0.21	0.20
Methionine+cystine	0.74	0.74	0.74	0.74	0.74	0.74
Threonine	0.70	0.70	0.70	0.70	0.70	0.70
Trypthopan	0.22	0.22	0.22	0.22	0.22	0.22
Valine	0.82	0.82	0.82	0.82	0.82	0.82
Arginine	1.11	1.11	1.11	1.11	1.11	1.11
Phenynlalanine	0.81	0.64	0.49	0.59	0.53	0.47
Tyrosine	0.57	0.45	0.35	0.55	0.47	0.37
Leucine	1.51	1.29	1.08	1.24	1.15	1.04
Isoleucine	0.65	0.65	0.65	0.66	0.65	0.65
Histidine	0.45	0.37	0.29	0.40	0.35	0.30
Glycine	0.99	0.79	0.61	0.81	0.66	0.50
Serine	0.86	0.69	0.53	0.64	0.58	0.50
Glycine+Serine	1.85	1.48	1.14	1.46	1.24	1.00

SBM, soybean meal; BSFLM, black soldier fly larvae meal.

1) Vitamin and mineral premix provide the following per kg of final diet: Vitamin A, 15,000.00 IU, Vitamin D_3_, 3,000.00 IU; Vitamin E, 24.00 mg; Vitamin K, 3.00 mg; Vitamin B_1_, 1.00 mg; Vitamin B_2_, 9.00 mg; Vitamin B_3_, 67.50; Vitamin B_5_, 18.60 mg; Vitamin B_6_, 3.00 mg; Vitamin B_9_, 1.50 mg; Vitamin B_12_, 0.03 mg.

**Table 2:** Effect of black soldier larvae meal in a low protein diet on growth performance of ducks

Parameter	Feed intake (g)	Body weight (g)	Weight gain (g)	ADG (g)	FCR
SBM-18	3,447.4^c^	1,438.6^e^	1,220.9^e^	38.2^e^	2.84^a^
SBM-16	3,098.1^b^	1,354.9^e^	1,136.6^e^	35.5^e^	2.73^a^
SBM-14	2,406.9^a^	952.9^b^	745.6^b^	23.0^b^	3.23^b^
BSFLM-18	3,062.9^b^	1,222.3^d^	1,005.0^d^	31.4^d^	3.05^ab^
BSFLM-16	2,791.2^b^	1,086.6^c^	868.8^c^	27.2^c^	3.21^b^
BSFLM-14	2,240.2^a^	788.3^a^	607.5^a^	19.0^a^	3.69^c^
SEM	117.33	39.20	39.10	1.20	0.11
CP Level					
18	3,255.2^c^	1,330.5^c^	1,112.9^c^	3,255.2^c^	2.95^a^
16	2,944.6^b^	1,220.8^b^	1,002.7^b^	2,944.6^b^	2.97^a^
14	2,323.6^a^	870.6^a^	676.5^a^	2,323.6^a^	3.46^b^
SEM	82.93	27.70	27.63	82.93	0.08
Protein source					
SBM	2,984.1	1,248.9	1,034.4	2,984.1	2.93
BSFLM	2,698.1	1,032.4	827.1	2,698.1	3.32
SEM	67.8^a^	22.6	22.6	67.8^a^	0.062
p-value					
CP level	<0.01	<0.01	<0.01	<0.01	<0.01
Protein source	<0.01	<0.01	<0.01	<0.01	<0.01
CP×protein source	ns	ns	ns	ns	ns

ADG, average daily gain; FCR, feed conversion ratio; SBM, soybean meal; SEM, standard error of mean; BSFLM, black soldier fly larvae meal; CP, crude protein; ns, non-significant.

a–e Values within a column with no common superscripts differ significantly (p<0.05).

**Table 3:** Effect of black soldier larvae meal in a low protein diet on blood biochemical profile of ducks

Parameter	Total protein (g/dL)	Glucose (mg/dL)	Urea (mg/dL)	Uric acid (mg/dL)	Calcium (g/dL)	Triglyceride (mg/dL)	Cholesterol (g/dL)	LDL (g/dL)	HDL (mg/dL)	Albumin (mg/dL)	Phospate (g/dL)
SBM-18	3.14^ab^	132.77^ab^	9.64	6.14	14.50	58.60^a^	142.40^a^	70.62^a^	52.04^a^	1.43^b^	9.58^a^
SBM-16	3.38^b^	125.80^ab^	9.08	6.23	14.89	60.07^a^	169.90^ab^	74.45^ab^	69.16^b^	1.63^b^	9.93^a^
SBM-14	2.76^a^	95.07^a^	8.30	6.26	14.41	76.77^b^	179.33^b^	99.67^c^	77.14^b^	1.42^b^	21.52^c^
BSFLM-18	2.80^a^	106.83^ab^	9.13	6.19	13.99	56.43^a^	197.70^b^	92.93^abc^	72.63^b^	1.04^a^	9.15^a^
BSFLM-16	2.89^a^	152.03^b^	9.06	6.29	13.77	73.08^b^	196.73^b^	97.52^bc^	77.75^b^	1.39^b^	15.94^b^
BSFLM-14	3.14^ab^	130.63^ab^	9.03	6.03	14.48	79.30^b^	254.03^bc^	122.77^d^	80.10^b^	1.45^b^	10.20^a^
SEM	0.13	10.48	0.46	0.18	0.82	3.75	11.01	7.37	5.50	0.07	1.02
CP Level											
18	2.97	119.80	9.38	6.17	14.25	57.51^a^	170.05^a^	81.77^a^	62.33^a^	1.24^a^	9.37^a^
16	3.13	138.92	9.07	6.26	14.33	66.57^b^	183.31^a^	85.99^a^	73.46^b^	1.51^b^	12.94^b^
14	2.95	112.85	8.66	6.15	14.45	78.03^c^	216.68^b^	111.22^b^	78.62^b^	1.43^b^	15.86^c^
SEM	0.09	10.04	0.32	0.13	0.58	2.66	7.81	5.23	3.91	0.05	0.72
Protein source											
SBM	3.09	117.88	9.00	6.21	14.60	65.14	163.88	81.58	66.11	1.49	13.68
BSFLM	2.94	129.83	9.07	6.17	14.08	69.60	216.15	104.41	76.83	1.29	11.77
SEM	0.08	8.20	0.26	0.11	0.47	2.17	6.38	4.29	3.18	0.04	0.58
p-value											
CP level	ns	ns	ns	ns	ns	<0.01	<0.01	<0.01	<0.05	<0.01	<0.01
Protein source	ns	ns	ns	ns	ns	ns	<0.01	<0.01	<0.05	<0.01	<0.05
CP×protein source	<0.01	ns	ns	ns	ns	ns	<0.01	<0.01	<0.05	<0.05	<0.01

LDL, low density lipoprotein; HDL, high density lipoprotein; SBM, soybean meal; BSFLM, black soldier fly larvae meal; SEM, standard error of mean; CP, crude protein; ns, non-significant.

a–d Values within a column with no common superscripts differ significantly (p<0.05).

**Table 4:** Effect of black soldier larvae meal in a low protein diet on morphology of jejunum of ducks

Parameter	Villus height (μm)	Crypt dept (μm)	VH:CD
SBM-18	959.5^c^	264.0^c^	3.52^a^
SBM-16	864.7^bc^	235.4^bc^	4.23^ab^
SBM-14	798.9^b^	255.6^bc^	3.89^ab^
BSFLM-18	670.6^a^	163.7^a^	4.09^ab^
BSFLM-16	678.0^a^	213.9^abc^	4.25^ab^
BSFLM-14	884.8^bc^	204.7^ab^	4.63^b^
SEM	36.03	17.30	0.30
CP level			
18	815.1	213.9	3.81
16	771.4	224.7	4.24
14	841.9	230.1	4.26
SEM	25.53	12.22	0.20
Protein source			
SBM	874.4	251.7	3.88
BSFLM	744.5	194.1	4.33
SEM	20.83	9.98	0.16
p-value			
CP level	ns	ns	ns
Protein source	<0.01	<0.01	ns
CP×protein source	<0.01	ns	ns

VH, villus height; CD, crypt dept; VH:CD, ratio villus height:crypt dept; SBM, soybean meal; BSFLM, black soldier fly larvae meal; SEM, standard error of mean; CP, crude protein; ns, non-significant.

a–c Values within a column with no common superscripts differ significantly (p<0.05).

**Table 5:** Effect of black soldier larvae meal in a low protein diet on gastrointestinal development of ducks

Parameter	Relative weight (g/kg)^1)^							Length (cm)						
	
	Proventriculus	Gizzard	Duodenum	Jejunum	Ileum	Caecum	Large intestine	Proventriculus	Gizzard	Duodenum	Jejunum	Ileum	Caecum	Large intestine
SBM-18	2.67^a^	30.17^a^	3.29^a^	6.62^a^	6.38^a^	1.38^b^	1.52^a^	4.90^bc^	7.02^b^	26.67^ab^	57.78	54.94^a^	26.00	8.75^c^
SBM-16	3.25^ab^	35.21^ab^	3.47^a^	9.35^b^	8.69^b^	1.59^b^	1.55^a^	5.35^c^	6.87^b^	27.12^b^	57.00	62.43^b^	28.23	8.60^c^
SBM-14	4.69^c^	42.22^c^	4.74^b^	11.39^cd^	9.69^b^	2.35^c^	2.85^b^	4.20^a^	5.75^a^	24.27^a^	54.85	56.75^ab^	26.33	6.90^a^
BSFLM-18	2.95^ab^	31.23^a^	3.23^a^	9.16^b^	8.88^b^	0.94^a^	1.72^a^	4.67^ab^	6.10^a^	25.67^ab^	57.67	58.00^ab^	25.83	8.33^bc^
BSFLM-16	3.17^ab^	32.08^ab^	3.55^a^	9.74^bc^	6.85^a^	1.64^b^	1.82^a^	4.38^ab^	5.85^a^	23.63^a^	56.74	58.38^ab^	28.57	8.30^bc^
BSFLM-14	3.87^bc^	37.48^bc^	4.88^b^	11.76^d^	9.52^b^	2.31^c^	2.01^a^	4.46^ab^	5.83^a^	24.90^a^	58.03	57.62^ab^	28.97	7.20^ab^
SEM	0.28	1.76	0.18	0.57	0.48	0.08	0.16	0.19	0.25	0.86	2.11	2.00	1.38	0.38
CP level														
18	2.81^a^	30.70^a^	3.26^a^	7.89^a^	7.63^a^	1.16^a^	1.63^a^	4.78^b^	6.56^b^	26.17	57.72	56.47	25.92	8.54^a^
16	3.21^a^	33.64^a^	3.51^a^	9.55^b^	7.76^a^	1.61^b^	1.69^a^	4.86^b^	6.36^ab^	25.37	56.87	60.40	28.39	8.45^b^
14	4.28^b^	39.85^b^	4.81^b^	11.57^c^	9.61^b^	2.33^c^	2.43^b^	4.33^a^	5.79^a^	24.58	56.44	57.19	27.65	7.05^b^
SEM	0.20	1.25	0.13	0.40	0.34	0.06	0.12	0.14	0.18	0.61	1.50	1.42	0.98	0.27
Protein source														
SBM	3.54	35.92	3.75	9.12^a^	8.25	1.77	1.98	4.82	6.55	26.22	56.54	58.04	26.85	8.08
BSFLM	3.33	33.98	3.85	10.22^b^	8.41	1.63	1.85	4.50	5.91	24.65	57.48	57.99	27.80	7.94
SEM	0.16	1.02	0.10	0.33	0.28	0.05	0.10	0.11	0.15	0.50	1.22	1.16	0.80	0.22
p-value														
CP level	<0.01	<0.01	<0.01	<0.01	<0.01	<0.01	<0.01	<0.05	<0.05	ns	ns	ns	ns	<0.01
Protein source	ns	ns	ns	<0.05	ns	ns	ns	ns	<0.01	ns	ns	ns	ns	ns
CP×protein source	ns	ns	<0.01	ns	<0.01	<0.05	<0.01	<0.05	ns	ns	ns	ns	ns	ns

SBM, soybean meal; BSFLM, black soldier fly larvae meal; SEM, standard error of mean; CP, crude protein; ns, non-significant.

1) Relative weight of slaughtered body weight (BW).

a–d Values within a column with no common superscripts differ significantly (p<0.05).

**Table 6:** Effect of black soldier larvae meal in a low protein diet on litter composition of ducks

Parameter	DM (%)	OM (%)	Nitrogen (%)	NH_3_ (mg/g)	pH
SBM-18	28.468	21.910	2.028^c^	2.420^b^	7.89^b^
SBM-16	28.922	22.184	1.773^bc^	2.242^ab^	7.65^ab^
SBM-14	27.702	21.164	1.239^a^	2.133^ab^	7.38^a^
BSFLM-18	28.080	21.022	1.603^ab^	2.317^b^	7.83^b^
BSFLM-16	28.610	21.776	1.347^ab^	2.004^ab^	7.57^ab^
BSFLM-14	26.554	20.240	1.210^a^	1.685^a^	7.52^ab^
SEM	1.52	1.18	0.14	0.18	0.13
CP level					
18	28.274	21.466	1.816^c^	2.37^b^	7.86^c^
16	28.766	21.980	1.560^b^	2.12^ab^	7.61^b^
14	27.128	20.702	1.225^a^	1.91^a^	7.45^a^
SEM	1.07	0.84	0.95	0.13	0.09
Protein source					
SBM	28.364	21.753	1.680	2.265	7.64
BSFLM	27.748	21.013	1.387	2.002	7.64
SEM	0.88	0.68	0.08	0,11	0.08
p-value					
CP level	ns	ns	<0.01	0.08	<0.05
Protein source	ns	ns	ns	ns	ns
CP×protein source	ns	ns	ns	ns	ns

DM, dry matter; OM, organic matter; NH_3_, ammonia; SBM, soybean meal; BSFLM, black soldier fly larvae meal; SEM, standard error of mean; CP, crude protein; ns, non-significant.

a–c Values within a column with no common superscripts differ significantly (p<0.05).
